# The genus *Cahara* Ghauri, 1978 of China (Hemiptera, Heteroptera, Pentatomidae, Halyini) with descriptions of two new species

**DOI:** 10.3897/zookeys.319.4275

**Published:** 2013-07-30

**Authors:** Zhong-hua Fan, Guo-qing Liu

**Affiliations:** 1 Institute of Entomology, Nankai University, No. 94 Weijin Road, Nankai District, Tianjin 300071, China

**Keywords:** Hemiptera, Heteroptera, Pentatomidae, *Cahara*, China, new species

## Abstract

*Cahara* Ghauri from China with three species is reviewed here. Two of them, *Cahara incisura* **sp. n.** and *Cahara nodula* **sp. n.** are described here. Key to the three Chinese species, habitus photographs and illustrations of genitalia are also provided. All examined materials including the types of three species mentioned are deposited in the Institute of Entomology, Nankai University, Tianjin, China (NKUM).

## Introduction

Ghauri (1978) erected a new genus *Cahara* of Halyini to accommodate three species formerly belonged to genus *Dalpada* Amyot & Serville, 1843, and the other six new species. Later in 1986, Zheng and Liu (1986) reported one new species *Cahara tibetana* found in China.

Xu and Zheng (1993) discussed the phylogenetic relationships of the nine genera of *Dalpada*-group including *Cahara* based on 18 morphological characters. Hasan and Kitching (1993) make a cladistic analysis of 21 tribes in Pentatomidae, and suggest a monophyletic clade as “megarrhamphine tribal-group” to accommodate Halyini, Megarrhamphini, Tetrodini and Phyllocephalini. Wall (2004) also states that the Halyini is not monophyletic. Memon et al. (2011) make a phylogenetic analysis using 61 morphological characters from 31 genera of south Asian Halyini, and the single most parsimonious tree shows that *Ameridalpa* Ghauri, 1982 is the sister group to *Cahara*, while the bootstrap support value is under 50%. Later Barão et al. (2012) point out that the analysis of Memon et al. (2011) has some under-developed aspects.

Here we do not discuss the status of *Cahara* in Halyini, since both the monophyly and definition of Halyini are doubted. Memon et al. (2011) only define the south Asian Halyini, and indicate that the inclusions in the Halyini of many genera from other parts of the world is under discussion, but *Cahara* has always been placed in Halyini (Ghauri 1978, Zheng and Liu 1986, Xu and Zheng 1993, Memon 2002, Rider 2006, Memon et al. 2011). In this paper two new species from China are described. So far totally twelve species of *Cahara* are recorded.

## Materials and methods

Male genitalia were illustrated after treatment with warm 2% NaOH solution for about 30–50 min, while female genitalia were illustrated directly. Photographs of both dorsal and ventral habitus were made using a Nikon SMZ1000. All measurements are in millimeters. All the studied specimens including the examined types are deposited in the Institute of Entomology, Nankai University, Tianjin, China (NKUM). The terminology of aedeagus follows Konstantinov and Gapon (2005), pygophore follows Schaefer (1977), female genitalia follows Schaefer (1967)

## Taxonomic part

### Cahara

Ghauri, 1978

http://species-id.net/wiki/Cahara

Cahara Ghauri 1978: 163; Rider 2006: 305; Xu and Zheng 1993: 18; Memon 2002: 117; Memon et al. 2011: 1049.

#### Type species.

*Dalpada brevivitta* Walker, 1867 by original designation.

#### Key to Chinese species of *Cahara*

**Table:** 

1	Lateral margin of each mandibular plate with an angular process before eye (Figs 3a–b); ventral margin of male pygophore with two mesial processes originating from one stem (Fig. 29)	*Cahara tibetana* Zheng & Liu, 1986
–	Lateral margin of each mandibular plate without any angular process before eye (Figs 1a–b, 2a–c); ventral margin of male pygophore without above processes	2
2	Apex of clypeus broad, mandibular plates not convergent at the apex (Figs 2a–c); humeral angles distinctly elevated (Fig. 5); rostrum passing beyond the middle of the 4^th^ sternum; ventral margin of male pygophore without process (Fig. 21)	*Cahara nodula* sp. n.
–	Apex of clypeus narrow, mandibular plates convergent at the apex (Figs 1a–b); humeral angles not elevated (Fig. 4); rostrum reaching the middle of 3^rd^ sternum; ventral margin of male pygophore with two lateral separated processes (Fig. 13)	*Cahara incisura* sp. n.

### Cahara incisura

Fan & Liu sp. n.

urn:lsid:zoobank.org:act:0ABF44CA-EE92-47D2-8864-68409E204BEA

http://species-id.net/wiki/Cahara incisura

Figs 1a–1b , 4 , 7–8 , 13–20

#### Type material.

**Holotype** male, pinned, **CHINA: Sichuan Province:** Mianning County, Liangshan Prefecture, 29. VIII. 2008, Kai DANG leg. **Paratypes**: all pinned, **CHINA: Sichuan Province:** 1 female, same data as holotype; 1 male, with genitalia in a separate microvial, same data as holotype.

#### Diagnosis.

Rostrum reaching the middle of 3^rd^ sternum, pronotal humeral angles not elevated upwards, apical meeting trend of mandibular plates are all similar to *Cahara tibetana*. But *Cahara tibetana* has a distinct angular process before each eye along the lateral margin of mandibular plate, mandibular plates about equal to or slightly longer than clypeus. While in this new species, mandibular plates are always longer than the clypeus, lateral margins of head sinuate and with no angular process before eye.

**Body size** Male, length 16.0mm, width between humeral angles 8.0mm. Female, length 17.0mm, width between humeral angles 8.5mm.

#### Description.

**Color and puncturing.** Dorsum fuscous, darkly and thickly punctured, with several obscure patches formed by dense punctures: four or five longitudinal strips on the pronotum, five on the scutellum (one short oblique strip near each arcuate callus behind the fovea of scutellar basal angle, one patch on central disk, two short longitudinal stripes at the level of the posterior apices of frena), two or three patches on each corium. Scutellar apex paler and punctures finer. Calli on the anterior disk of pronotum ochraceous with punctures in the middle. Humeral angles piceous, tips a little pale, with several transverse furrows and wrinkles on the dorsal base. Hemelytral membrane fuliginous, except apices of veins paler. Head beneath black, except buccula and one obscure strip behind each antenniferous tubercle ochraceous. Thoracical pleura thickly and darkly punctured, each episternum with an ochraeous, laevigate and arcuate fascia distally. Mesosternum black strips laterally. Legs ochraceous, with irregular brown spots, tibiae paler in the middle third and darker in the apical third, first two tarsal segment and apex of the third one white dorsally. Ventral abdomen smooth at center, punctures gradually getting denser laterally. Middle third of each laterotergite with a transverse brown impunctate stripe.

**Figures 1–3.**
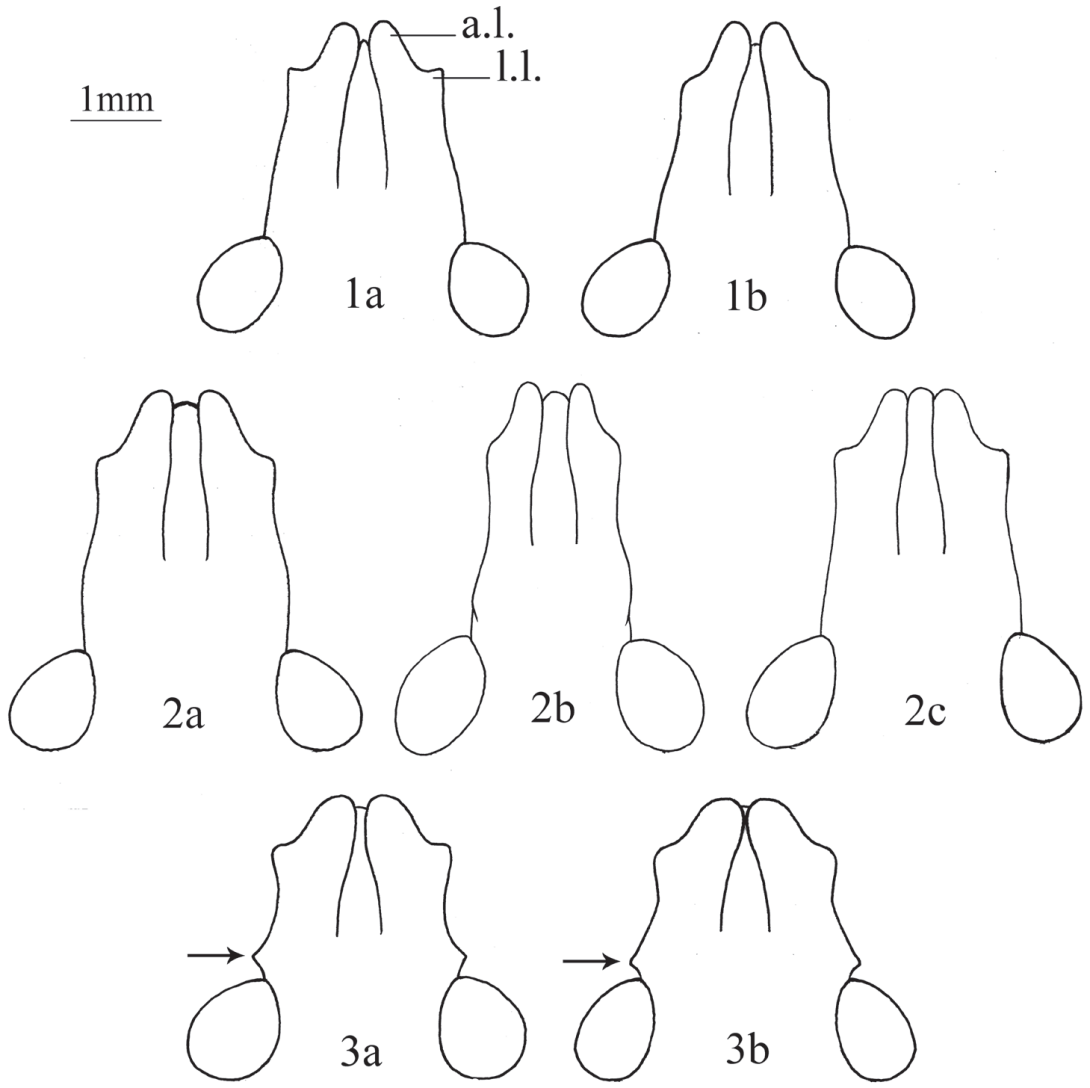
Heads in dorsal view. **1a–b**. *Cahara incisura* sp. n. (**a** holotype, **b** paratype). **2a–c** *Cahara nodula* sp. n.(**a** holotype, **b** paratype, **c** paratype) **3a–b** *Cahara tibetana* (**a** holotype, **b** allotype). (a.l. apical lobe of mandibular plate, l.l. lateral lobe of mandibular plate).

**Structure. Head.** Mandibular plates longer than clypeus, apices with meeting trend but still separated, forming an incision before clypeus. Lateral lobes of mandibular plates are found angulate in the male holotype and the female paratype (Fig. 1a), but obtuse in the male paratype (Fig. 1b). Buccula with anterior angle not produced, gradually evanescent posteriorly. Antennae ochraceous, darker to the end, antennomere I paler except each lateral side, base of antennomere IV and basal third of antennomere V stramineous, IV> V>III >II>I in length. Rostrum reaching the middle of 3^rd^ sternum, apex of 1^st^ segment equal to the posterior end of buccula.

**Thorax.** Pronotum with anterior margin slightly convex in the middle, anterior angle produced laterad, anterolateral margins concave, crenulate along the anterior half, crenulation getting weaker posteriorly. Humeral angles horn-like, apices obtuse, slightly produced and not elevated upwards. Hemelytral membrane longer than the abdominal end. Peritreme groove shaped according to Kment and Vilímová (2010), which is narrow, long, curved, apex rounded, median furrow is well developed in most of its length. A narrow and long carina along the midline of mesosternum.

**Abdomen.** Connexiva exposed broadly, posterior angles sharp and produced. Mesial groove on ventral side not distinct.

**Male genitalia.** Ventral rim of pygophore with two separated processes on both lateral sides. Suspensory apodeme and infoldings of lateral rims developed. Paramere L–shaped, stem with a short basal process, apex of blade obtuse without any distinct process. Phallotheca cylindric, with a mesial process on the base of ventral side. Aedeagus with a pair of dorsal conjunctival processes, sclerotized and fingerlike, a trifurcate membraneous conjunctival lobe, a pair of ventral conjunctival processes, slightly sclerotized. Median penial plates oblong and narrow, about as long as the protrudent vesica.

**Female genitalia.** Paler in color, punctured on gonocoxites I and paratergites VIII, punctures on gonocoxites I finer. Mesial margins of gonocoxites I narrowly black, meeting each other along the basal halves, lateral margins of the fingerlike processes not vertical. Gonocoxite II with a transverse tumescent beam full of setae. Paratergits IX obtuse apically, slightly passing beyond the posterior margin of 8^th^ sternum. Paratergites VIII not protrudent apically.

**Figures 4–6.**
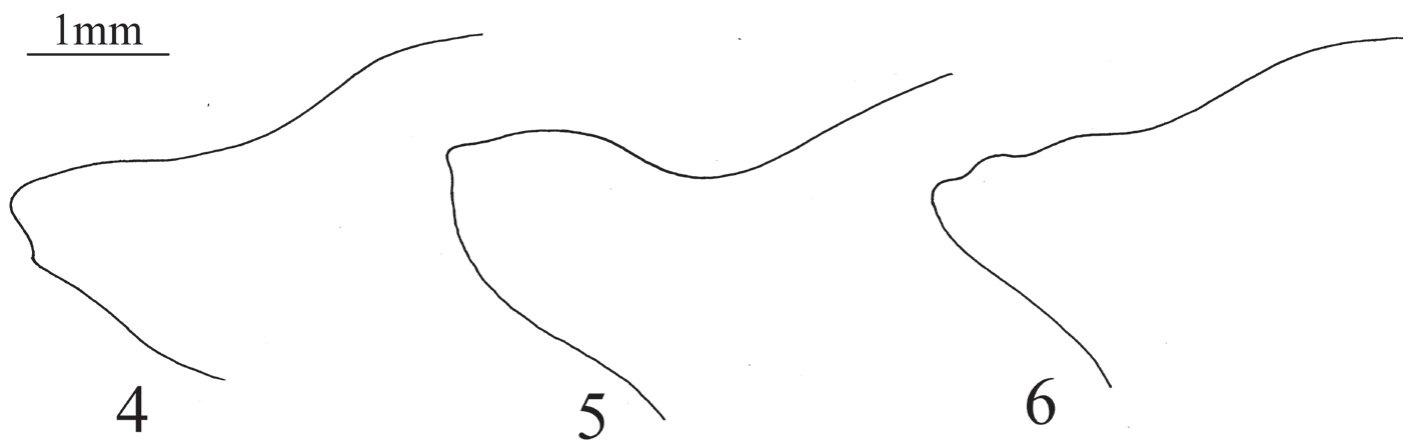
Right humeral angles in cephalic view. **4** *Cahara incisura* sp. n. **5** *Cahara nodula* sp. n. **6** *Cahara tibetana*.

**Figures 7–12.**
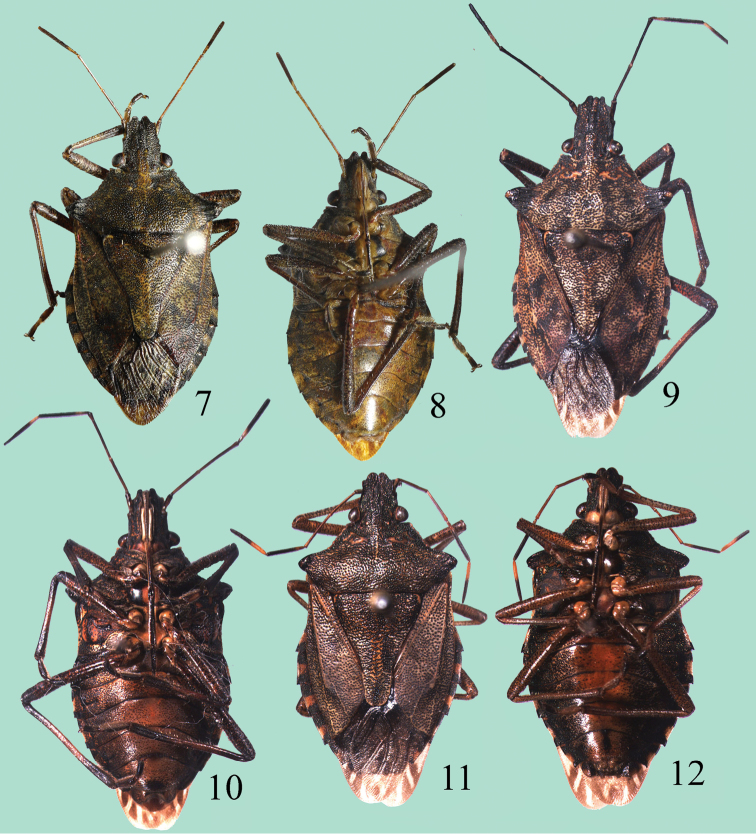
*Cahara* spp. **7–8** *Cahara incisura* sp. n., holotype **9–10** *Cahara nodula* sp. n., holotype **11–12** *Cahara tibetana*, allotype.

**Figures 13–20.**
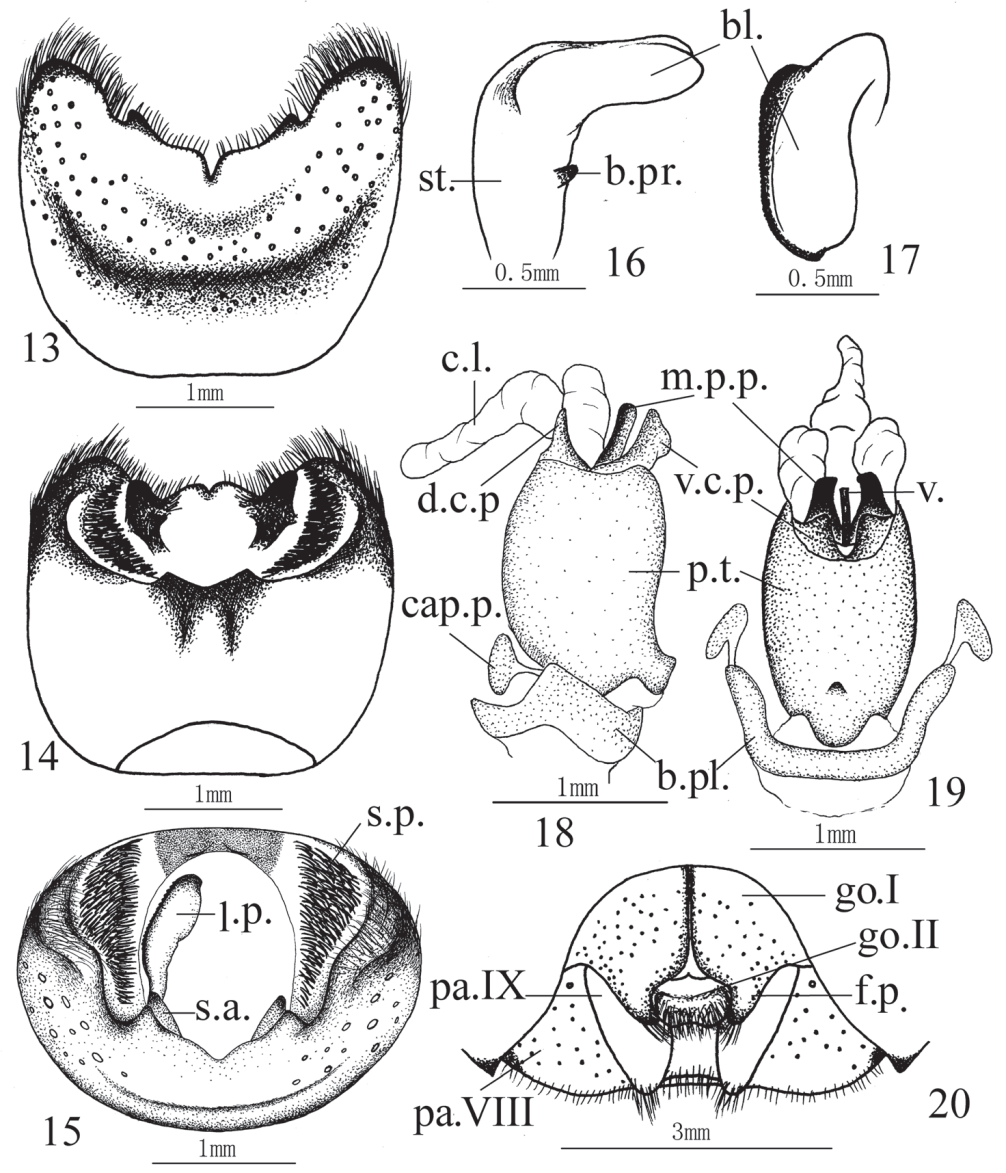
*Cahara incisura* sp. n. **13–15** Pygophore (**13** ventral view, **14** dorsal view, **15** caudal view) **16–17** Paramere (**16** lateral view, **17** caudal view) **18–19** Aedeagus (**18** lateral view, **19** ventral view) **20** Female genitalia. (bl. blade, b.pl. basal plate, b.pr. basal process, cap.p. capitate process, c.l. conjunctival lobe, d.c.p. dorsal conjunctival process, f.p. fingerlike process, go.I gonocoxite I, go.II gonocoxite II, l.p. left paramere, m.p.p. median penial plate, pa.VIII paratergite VIII, pa.IX paratergite IX, p.t. phallotheca, s.a. suspensory apodeme, s.p. setal patch, st. stem, v. vesica, v.c.p. ventral conjunctival process)

#### Etymology.

The species name, *incisura*, refers to the longer mandibular plates that always leave an incision before clypeus. It’s feminine.

#### Distribution.

Southwest China (Sichuan)

### Cahara nodula

Fan & Liu sp. n.

urn:lsid:zoobank.org:act:EFEF49E2-14C7-41AF-9901-FB3F60AAC9FE

http://species-id.net/wiki/Cahara_nodula

Figs 2a–2c , 5 , 9–10 , 21–28

#### Type material.

**Holotype** male, pinned, **CHINA: Yunnan Province:** Xiang Mount., 5. VIII. 1979, Huan–guang ZOU leg. **Paratypes**: all pinned, **CHINA: Yunnan Province:** 1 female, same place and collector as holotype, 2. VIII. 1979; 1 female, same place as holotype, 15. VIII. 1979, Zuo–pei LING leg.; 2 males (one with genitalia in a separate microvial), 1 female, Kunming, VII. 1957; 1 female, same data as above except date, IV. 1986; 1 female, same data as above except date, V. 1986; 1 female, same data as above except date, VII. 1986; 1 male, same data as above except date, 17. VI. 1988; 1 male, Anning City, 12. V. 1988, Yun–xu WANG leg.; 1 female, Wushan Town, Mile County, alt. 2000m, 20. V. 1979, Guang–qiang YIN leg.; 1 male, with genitalia in a separate microvial, Santai Village, Dayao County, 13. VI. 1978; 1 female, Dayao County, VIII. 1980; 1 female, Dechang County, VI. 1958; 1 female, Weishan County, 4. VI. 1978; **CHINA: Guizhou Province:** 2 males, Huaxi District, Guiyang City, 23. V. 1987; 1 female, Changming Town, alt. 1050m, 9. IX. 2000, Chuan–ren LI leg.; 1 female, Fanjing Mount., alt. 1300m, 1. VIII. 2001, Wei–bing ZHU leg.

#### Diagnosis.

Humeral angles nodular and elevated upwards (Fig. 5), rostrum longer to pass beyond the middle of the 4^th^ sternum, mandibular plates without meeting trend apically, 1^st^ rostral segment passing beyond the posterior end of buccula, ventral rim of pygophore without any distinct processes (see discussion part).

#### Body size.

Male, length 16.0–18.0 mm, width between humeral angles 8.0–8.8 mm. Female, length 19.0–20.0 mm, width between humeral angles 9.0–10.0mm.

#### Description.

**Color and puncturing** Very similar to *Cahara incisura*, but with some differences: Punctures on dorsal head denser, while sparser and finer on the endocorium, pronotum with four or five longitudinal strips, laevigate parts of calli more distinct.

**Stucture. Head.** Mandibular plates about equal to clypeus or slightly longer than clypeus, apices porrect and having not convergent, both apical and lateral lobes obtuse distally, lateral margins before eye sinuate and without any distinct process. Apex of clypeus broadly exposed (Figs 2a–c). Antennae brown, antennomere I paler, with a longitudinal black strip laterally, apical two third of antennomere IV and apical half of antennomere V black, IV>III≥V>II>I in length. Buccula low, anterior angles pointed and protrudent, outer margins straight. Rostrum with 1^st^ joint extending beyond the buccula, apex reaching to the middle of 4^th^ sternum.

**Thorax.** Pronotum with anterior half depressed and posterior half tumescent, anterior margin broad, sinuate, slightly convex mesially, anterior angle small, angulate and produced laterad, anterolateral magins crenulate, humeral angles nodular, protrudent, elevated upwards. Scutellum longer than width, basal disk and longitudinal midline tumescent. Meso sternum flat with a mesial narrow carina. Peritreme similar to *Cahara incisura*. Hemelytral corium longer than scutellum, membrane extending beyond the abdominal end.

**Abdomen.** Connexiva exposed, posterior angles pointed and produced. Venter, from 3rd to 6th abdominal sternite, with a mesial shallow groove.

**Male genitalia.** Ventral rim of pygophore V–shaped excavated, sinuate along the margin but without distinct process. Suspensory apodeme and infoldings of lateral rims developed. Paramere L–shaped, stem broad with a small basal process, blade long with an apical process and a basal process, these two processes all directed caudad. Aedeagus with paired sclerotized dorsal conjunctival processes, a trifurcate membranous conjunctival lobe, and a pair of slightly sclerotized ventral conjunctival processes. Median penial plates oblong, apices obtusely angulate. Vesica slim, protrudent.

**Female genitalia.** Outer margins of gonocoxites I black, so are the apical halves of paratergites IX, pratergites VIII thickly punctured. Gonocoxites I strongly sinuate mesially, broadly and distinctly depressed in the middle of the lateral margins so the fingerlike processes bent dorsally and almost vertical. Apices of fingerlike processes reaching the apical third of paratergites IX. Gonocoxite II with a transverse tumescent beam. Paratergite IX base with a short oblique ridge, apex passing a little beyond the posterior margin of 8^th^ sternum. Paratergites VIII obtuse distally.

**Figures 21–28.**
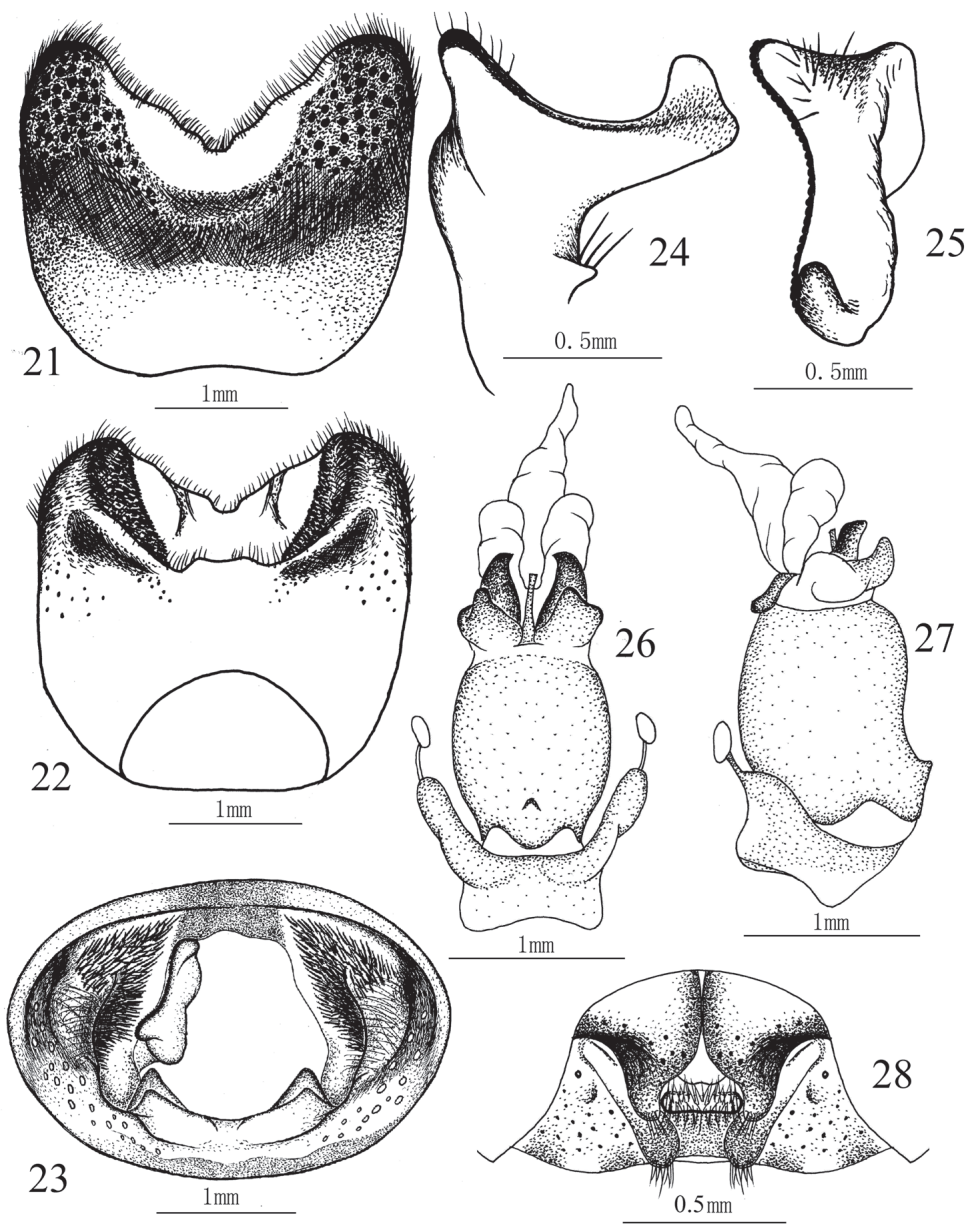
*Cahara nodula* sp. n. **21–23** Pygophore (**21** ventral view, **22** dorsal view, **23** caudal view) **24–25** Paramere (**24** lateral view, **25** caudal view) **26–27** Aedeagus (**26** ventral view, **27** lateral view). **28** Female genitalia.

#### Etymology.

The name, *nodula*, refers to the bulbous, distinct nodular humeral angles of pronotum. It’s feminine.

#### Distribution.

Southwest China (Guizhou, Yunnan)

### Cahara tibetana

Zheng & Liu, 1986

http://species-id.net/wiki/Cahara_tibetana

Figs 3a–b , 6 , 11–12 , 29–36

Cahara tibetana Zheng and Liu 1986: 163; Rider 2006: 305; Xu and Zheng 1993: 18.

#### Type material examined.

**Holotype**, male, pinned, with genitalia in a separate microvial, **CHINA: Xizang Autonomous Region**: Chayu County, alt. 1700m, 26. VI. 1978, Fa–sheng LI leg. **Allotype**, 1 female, pinned, same data as holotype.

#### Other material examined.

**CHINA: Xizang Autonomous Region:** 1 female, Dongjiu Nature Reserve, 21. IX. 2007, Fu–min SHI leg.; 1 female, Motuo County, alt. 800m, VIII. 1984.

#### Diagnosis.

See diagnosis of *Cahara incisura* sp. n. Besides, in this species, two processes on the ventral rim of pygophore connected basally, suspensory apodeme longer than those in the other two species, stem of paramere broad with two lateral margins not parallel. Gonocoxites I depressed along the lateral margin and fingerlike processes bent dorsally like *Cahara nodula*, but the transverse tumescent beam of gonocoxite II shorter in this species.

#### Distribution.

Southwest China (Xizang)

**Figures 29–36.**
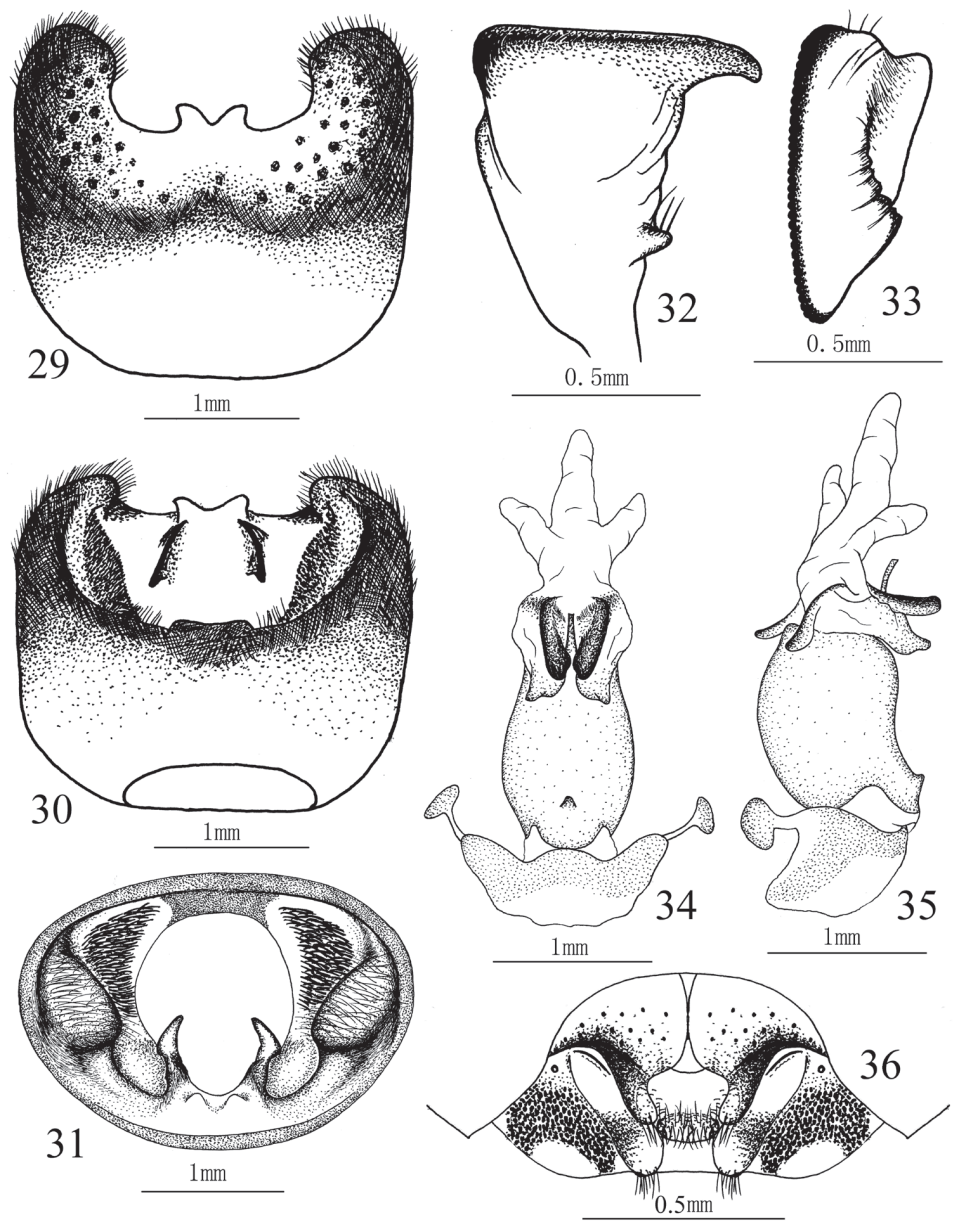
*Cahara tibetana* Zheng & Liu. **29–31** Pygophore (**29** ventral view, **30** dorsal view, **31** caudal view) **32–33** Paramere (**32** lateral view, **33** caudal view) **34–35** Aedeagus (**34** ventral view, **35** lateral view) **36** Female genitalia.

## Discussion

Ghauri (1978) gives both the external and internal diagnostic characters of *Cahara* to distinguish it from *Dalpada* s.s. Some of them are cited and discussed as follows:

1. “Juga longer than tylus”. There are variations at least in *Cahara nodula* sp. n. and *Cahara tibetana*, but it’s true that “juga” is never found shorter than “tylus”.

2. “Pygophore, ventral margin with more or less deep concavity with a pair of median lobes.” The mesial concavity is also present in many species of *Dalpada*. But the paired median processes play a vital role to identify the genus. It happens in most of the species of *Cahara*, but not in *Cahara nodula* sp. n.

3. “Female genitalia: … first valvifer produced posteriorly.” In our views, this finger-like process elongated from the posterior apex of gonocoxite I is the most effective diagnostic character to distinguish *Cahara* from the other genera of Pentatominae in which it’s rare. *Izharocoris* Afzel & Ahmad, 1981 (Pentatomidae: Halyini) is the other related genus that some of its species share this character. But it’s different for having paramere with both the inner and the outer processes (Memon 2002) while only one inner basal process in *Cahara* (Figs 16, 24, 32). We placed the two new species in *Cahara* mainly basing on this point.

4. Ghauri (1977) indicates that *Sarju* is closely related to *Cahara*, and “the absence of median lobes in the concave ventral margin of pygophore” in *Sarju* is diagnostic. But as an exception, *Cahara nodula* sp. n. has no such “median lobes” either. The other useful diagnostic character is the antennomere II “distinctly bowed and appreciably swolen at apex” in *Sarju* instead of *Cahara* (Ahmad & Afzal 1984).

Those twelve *Cahara* spp. (nine species recorded by Ghauri (1978), three others here) are very similar by outlook. The useful characters to distinguish them are: the shapes of ventral rim of male pygophore, paramere distal apex, finger-like processes of gonocoxites I. By comparing the ventral rims of pygophore, the two new species *Cahara incisura* and *Cahara nodula* can be easily recognized from the five species of which male specimens are described in Ghauri (1978) (*Cahara brevivitta* (Walker), *Cahara murreeana* (Ghauri), *Cahara montana* (Ghauri), *Cahara kightleyi* (Ghauri), *Cahara jugatoria* (Lethierry)). While the other four species with only females described can be excluded by female genitalia characters (1. *Cahara confusa* (Distant), mandibular plates meeting in front of clypeus, paratergites IX with inner margins bisinuate; 2. *Cahara chaubattia* (Ghauri), gonocoxites I with inner margins entirely separated from each other; 3. *Cahara bhowaliana* (Ghauri), processes of gonocoxites I much longer, passing beyond gonocoxites II; 4. *Cahara metallica* (Ghauri), finger-like processes of gonocoxites I very narrow, short and sharp, gonoxites II exposed widely. All the above distinctive characters are not existing in both *Cahara incisura* sp. n. and *Cahara nodula* sp. n.)

**Figure 37.**
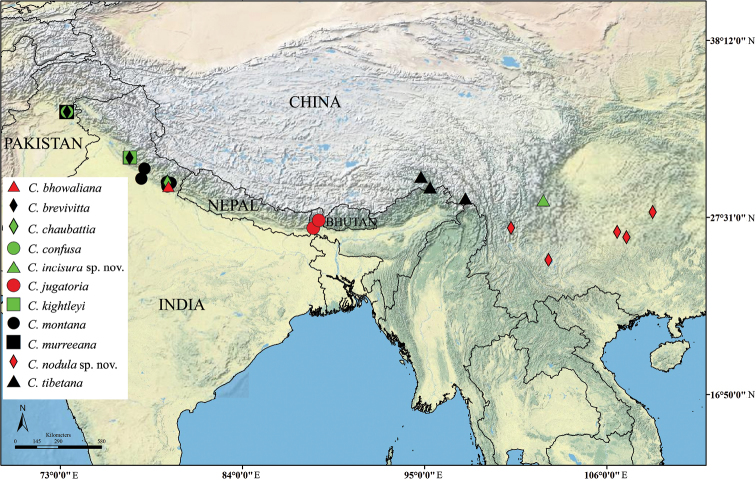
Geographical distribution of eleven *Cahara* spp.

According to Ghauri (1978), the genus *Cahara* occurs in the subhimalayan region of India, Pakistan and Nepal, while the three species from southwest China are obviously from the northern Himalayan region. Till now, no distribution overlap between the southern and northern Himalayan species was found. We tried to make a distribution map (Fig. 37) of the twelve species of *Cahara* based on the published distribution information (Ghauri 1978, Zheng and Liu 1986), but only eleven are showed on the map. The only two localities of *Cahara metallica* as well as several other localities, mentioned by “*” and “?” in Table 1, are excluded, because they look too obscure for mapping.

**Table 1.** Distribution information of twelve *Cahara* species.

**Species**	**Locality**	**Geographic coordinates**
*Cahara bhowaliana*	Bhowali, India	29.3833°N, 79.5167°E
*Cahara brevivitta*	Simla, India	31.1046°N, 77.1734°E
	Murree, Pakistan	33.9065°N, 73.3937°E
	*Koozagali, Pakistan	?
	*Cahar (Bowring), India	?
*Cahara chaubattia*	Chaubattia, India	29.6137°N, 79.4563°E
*Cahara confusa*	Murree, Pakistan	33.9065°N, 73.3937°E
*Cahara incisura* sp. n.	Mianning, China	28.5496°N, 102.1770°E
*Cahara jugatoria*	Kurseong, India	26.8800°N, 88.2783°E
	Gantok,Sikkim, India	27.3389°N, 88.6065°E
	*Himalayas Terai, India	?
*Cahara kightleyi*	Simla, India	31.1046°N, 77.1734°E
	Mashobra, India	31.1296°N, 77.2283°E
*Cahara metallica*	*Hardwicke Bequest, ??	?
	*??, Nepal	?
*Cahara montana*	Roorkee, India	29.8543°N, 77.8880°E
	Nainital, India	29.3803°N, 79.4636°E
	Almora, India	29.5984°N, 79.6615°E
	Ranikhet, India	29.6434°N, 79.4322°E
	Mussoorie, India	30.4553°N, 78.0741°E
*Cahara murreeana*	Murree, Pakistan	33.9065°N, 73.3937°E
	Ghora gali, Pakistan	33.8874°N, 73.3620°E
*Cahara nodula* sp. n.	Xiang Mount., China	26.8910°N, 100.2160°E
	Anning City, China	24.9594°N, 102.4821°E
	Huaxi, China	26.3331°N, 107.1949°E
	Changming, China	27.8407°N, 108.7735°E
	Fanjing Mount., China	26.6474°N, 106.6301°E
*Cahara tibetana*	Chayu, China	28.6613°N, 97.4669°E
	Dongjiu, China	29.9601°N, 94.7792°E
	Motuo, China	29.3253°N, 95.3332°E

## Supplementary Material

XML Treatment for Cahara

XML Treatment for Cahara incisura

XML Treatment for Cahara nodula

XML Treatment for Cahara tibetana
